# News and Miscellany

**Published:** 1882-07

**Authors:** 


### NEWS AND MISCELLANY.

Professor David, Director of the Veterinary School at Bern, died recently.

Royal College of Veterinary Surgeons.—At the annual meeting, Mr. George Fleming, F.R.C.V.S., was re-elected President of the Council for the third time, and Prof. A. Smith, Dean of Toronto Veterinary College, was made an honorary member.

Edinburgh Veterinary College. —The appointment of Professor of Physiology in the Dick Veterinary College having become vacant through the translation of Dr. Cunningham to Dublin, the Town Council of Edinburgh on Tuesday elected Dr. James (recently appointed Assistant Physician to the Royal Infirmary) to the Chair of Physiology in the College. The other candidate, Mr. Johnson Symington, M.B., had previously withdrawn his application, so that there was no contest.

Royal Agricultural Society.—The annual meeting of the members took place at the office, Hanover Square. The report submitted showed that the society had a total of 8,080 members, being an increase of 101 since the last annual meeting. The funded property of the society has been increased by the investment of £3,000, and now stood at £18,428 new Three Per Cents. The Duke of Richmond and Gordon was elected president for the ensuing year; twenty-five retiring members of the Council were elected, and Lord Evelyn was elected in the place of the Earl of Leicester.

Cæsarian Section in a Cow.—Dr. William A. Jackman sends us the following account of the operation: The cow belonged to Mr. J——, and was unable to give birth to the calf, which afterwards proved to be a very large one. The cow died, and a few minutes after Mr. J. discovered the calf was alive, and it occurred to him that the calf might be saved. The operation was performed with a pocket knife, the calf saved, and is now doing well; I have seen it myself. The most singular point in this case is that it should have been done by a *farmer* with a pocket knife, who perhaps had never heard of such a thing before, at least says he never did in the human race.

Decrease of Sheep in England.—It is stated that, at the present time, the stock of sheep in England is considerably smaller—some estimates put it at 2,000,000—than it has been for many years past, and the primary cause is said to be the liver rot, which caused serious losses in sheep in the midland counties during the wet season last year. It is believed that it will be two or three years before the great loss is made good; but, no doubt, the present lambing season, which is most encouraging, will have a tendency to improve the present state of affairs. Farmers give very encouraging reports with respect to their lamb crop, many of them stating that their ewes are dropping couplets and triplets.—*Drover's Journal.*

Consumption of Horse Flesh.—An official report just published shows that, since the time when horse flesh was first retailed in Paris as an article of human food, the consumption of that delicacy has steadily increased. In 1875 the number of horses slaughtered for this purpose was 7,000, and this had risen to 9,000 in 1880, and again to 9,300 in 1881. Besides these there were sold at the forty establishments, exclusively devoted to carrying on this business, ten carcases of donkeys in the first mentioned year, 320 in the second, and 400 in the third. The estimated weight of horse flesh consumed in Paris last year was about 1,670 English tons, in addition to about eighteen tons of donkey flesh, without reckoning the offal, which is used in the making of sausages.—*Drover's Journal.*

A lecture on horses was delivered at Trinity College, Dublin, recently, by the Rev. J. P. Mahaffy, Professor of Ancient History in the University of Dublin. The reverend gentleman said that all the facts were in favor of the development of the horse, the rhinoceros, and the tapir from a common parent. It was doubtful whether there now existed in the world a real specimen of the original wild horse. Many people thought that what were now apparently wild horses in Upper Asia were only the descendants of tame horses that had been let loose. There was reason to believe that there yet roamed in the high and cold plains of inner Asia, two or three kinds of horses that had never been tamed. The domestic horse now in use was liable to panics. This had been supposed to be due to improper treatment and feeding; but observers of the horse which ran wild in America had seen that when herds of them smelt water they rushed to it with such violence that they killed each other. The first stage of domestication of the horse was the nomadic, in which the animal was only used for food.—*Drover's Journal.*

Horse-Cleaning Machine.—A novel machine has been exhibited at New York for cleaning horses. The "Lightning Horse-Cleaning Machine" is almost similar in appearance to a trapeze, and about the size of the trapeze rope. At the end of either arm is a hair brush of superior bristles. Steam power was used, and in action it is simply an "automaton man," reaching from the nostril to under the fetlock joint of the horse, over and to every part of the body. In rapidity, it works fifty times faster than a man, and in its motion this wonderful "universal joint" turns about the brush in every possible direction, describing the complete radius of a sphere of 360 degrees. The machine was seen in full operation by a correspondent, and horses were passing rapidly to it and through the grooming process. Some horses were cleaned in from one and a half to two minutes. The skin was found to be perfectly clean, and one application also highly improved the appearance of the coat.

Animals That Drink Rum.—It is to be hoped that all the temperance people have read of Jumbo's whiskey drinking during the trip from England, for it is quite time that the old theory that animals will not touch liquor should be swept away. But Jumbo is not the only four-footed beast that indulges in strong drink. Thousands of race-horses have taken a pint of whiskey or brandy before running, oxen have been dosed with rum before going into great dragging matches, hogs learn more easily to drink than boys do, tame bears will guzzle beer like so many capacious Germans, chickens will swallow rum-soaked corn as long as their "crops" will stretch, and bulldogs will cry—or whine—for any alcoholic liquor of which they have ever felt the effects. If, instead of talking twaddle about liquor, the temperance people would explain that the great risk in the use of any stimulant is that those who once have felt its exhilarating effects are sure to long for them again, and that all animal life, high and low, vulgar and refined, is alike in this respect, they would do more good and be less laughed at.

Ways in Which Milk May be Affected.—A summary of the different ways in which milk may become so affective as to transmit disease to human beings has been presented by Dr. Vacher: 1. The milk may be derived from a tuberculous cow. 2. It may be derived from a cow suffering from a specific epizootic disease. 3. It may be drawn from an inflamed udder. 4. It may have undergone chemical or fermentative change. 5. It may have become infected with the contagium of an animal disease. 6. It may have become infected with the contagium of a human disease. Two preventives of mischief are recommended to the consumer: 1. To examine the milk delivered to him and to reject it if it appears to be watered, or if it is streaky, ropy, blood-stained, or smells bad from any cause. 2. To insist upon all the milk received being thoroughly boiled. To these precautions may be added that of keeping the milk in covered vessels in closets where the atmosphere is clean and cool. Milk or butter, unless carefully preserved from contamination, will very soon play havoc with a sensitive constitution. The restaurant system of exposing butter to an atmosphere charged with all manner of abominations is highly reprehensible.

That Dogs Appreciate Medical Treatment is a fact that has been often attested. A story has lately been published illustrating this. 'One afternoon a little black-and-tan dog was found moaning piteously at the entrance of a hospital in East Concord street, Boston. The animal's apparent suffering attracted the attention of Dr. C. E. Hastings, one of the faculty, who made an examination, which revealed a bad compound fracture of the leg. The doctor set the bone, and gave the little sufferer such other treatment as the case required.

The Cemetery for Cats.—The burial-place for pet animals, dogs, cats, and little birds is emerging from the region of dreams. The prospectus of the Zoölogical Necropolis Association (limited), which lies before us, with its imposing array of patrons, directors, bankers, brokers, solicitors, and secretaries, shows that the scheme is being pushed in the orthodox commercial fashion, and any one who wishes to subscribe can purchase as many of the five thousand two-pound shares as his inclination leads him and his finances permit. The burial-ground is to be established "within a few miles of London," and, "if wished for, a tribute to their memory can be erected by those who love them." In due time we shall have a cats' undertaker setting up in business, but for the present it is sufficient to say that the offices of the Cats' Cemetery Company are at No. 27 Henrietta street, Cavendish Square, W., where all necessary information can be obtained by intending shareholders.

Bird's-Nest in a Horse's Tail.—Interesting cases have been from time to time recorded of extraordinary places selected by birds for habitation and nesting; and I take this opportunity of bringing to your notice a case which occurred w en I was in camp at Fort Napoleon, Conference Hill, Zululand, and which appears to me to be unique.

A gray gelding cob, bought about the end of June, 1879, at Wakkerstroom, from Mr. Fawcus, a government surveyor, whilst I was on special duty purchasing remount horses for the Cavalry Brigade, was noticed at the time of purchase to have a peculiar knotted condition of the tail. After arriving at its destination at Fort Napoleon, several days' march distant, it was placed on the flank of the troop of King's Dragoon Guards, to which it was told off. The next morning, after reveille, the non-commissioned officer in charge of the troop noticed a little dark-colored bird (known, I am told by our interpreter, as a weaver or bottletit) fly and conceal itself in the cob's tail just at the extremity of the dock. Shortly afterwards he saw it reappear, settle near some spilt forage in the picket lines, feed, and then return to its former hiding-place. This roused the curiosity of the non-com., who, accompanied by several of the men of his troop, examined the cob's tail, and there found a perfectly-formed bird's-nest, about three inches in diameter, and about six inches from top to base, beautifully lined with short chestnut-red hair, which, upon examination, I found to have been collected from the red transport oxen, and not from chestnut horses. The most striking thing which occurs to me is that the little bird must have accompanied the cob from Wakkerstroom, in the Transvaal, to our camp in Zululand, sufficient time not having elapsed since its arrival at the fort for so complete a nest to have been manufactured.

When I bought remount horses, I always immediately cut their tails short, so that I could easily distinguish them, when grazing during the day, from other horses belonging to civilians and others; and when squaring this cob's tail with the scissors I remarked to Capt. Becher, who was with me, that the tail was peculiarly matted and curled, and therefore very difficult to cut quickly. The cob was driven, with a string of others, by Zulus from Wakkerstroom to our camp, about five days' journey.

As a twig of any sort, to say nothing of a tree, is quite a rare thing to see in many parts of the Transvaal and Zululand, and as the nights are particularly cold, I can quite understand these tame little birds getting into the hair of a horse's tail for warmth when the animal was lying down, and, later on, taking it into their heads to make a nest in such comfortable quarters.

S. Longhurst, A.V.D., K.D. Guards (Meerut, Bengal).

—*The Field.*

To Keep Flies from Horses.—The *Indiana Farmer* advises its readers to procure a bunch of smartweed and bruise it to cause the juice to exude. Rub the animal thoroughly with the bunch of the bruised weed, especially on the legs, neck and ears. Neither flies or other insects will trouble him for twenty-four hours. The process should be repeated every day. A very convenient way of using it is to make a strong infusion by boiling the weed a few minutes in water. When cold it can be conveniently applied with a sponge or bush. Smartweed is found growing in every section of the country, usually in wet ground near highways.

The (Veterinary) Anatomist to His Dulcinea.—

I list as thy heart and ascending aorta
  Their volumes of valvular harmony pour,
And my soul from that muscular music has caught a
  New life 'mid its dry anatomical lore.

Oh! rare is the sound when thy ventricles throb
  In a systolic symphony measured and slow;
When the auricles answer with rythmical sob,
  As they murmur a melody wondrously low!

Oh! thy cornæ, love, has the radiant light
  Of the sparkle that laughs in the icicle's sheen,
And thy crystalline lens, like a diamond bright,
  Through the quivering frame of thy iris is seen.

And thy retina, spreading its lustre of pearl,
  Like the far-away nebula distantly gleams
From a vault of black cellular mirrors that hurl
  From their hexagon angles the silvery beams.

Ah! the flash of those orbs is enslaving me still,
  As they roll 'neath the palpebræ, dimly translucent,
Obeying in silence the magical will
  Of the oculo-motor, pathetic, abducent.

Oh! sweet is thy voice as it sighingly swells
  From the daintily-quivering chordæ vocales,
Or rings in clear tones through the echoing cells
  Of the antrum, the ethmoid and sinus frontales.

TURKISH BATHS FOR HORSES.—Dr. Isaac Hough, surgeon-in-charge of the Third Avenue Railroad stables, in which there are 2,000 or more horses, has been successfully treating the disabled ones by a system of Turkish and Russian baths. A space about as large as six or eight ordinary stalls has been tightly inclosed in a corner of the building, with thick board walls, floor and ceiling. The floor is covered with movable slats, and under these slats are steam pipes, by which the temperature can be run up to 100, 150 or 200 degrees, at the will of the operator. In another apartment are rows of gas-burners, for horses requiring treatment with hot air. In front of two of the stalls are small, square windows, each large enough for a horse to put his head through. These windows can be closed with board shutters, and they are also provided with heavy curtains, opening in the centre. When it is desirable to give a horse a very hot bath in a room whose temperature must be higher than the horse could breathe with comfort, one of the windows is opened, and the horse, with his body in the bath, puts his head out into the cool air. The curtains are then drawn tightly about his neck to prevent the heat from escaping, and the horse has all the luxury of a Turkish bath without the inconvenience of breathing the overheated air. Horses requiring treatment are left in the hot bath from twenty minutes to an hour, and are then washed with tepid water and thoroughly scrubbed. A horse, on coming out of the bath, is taken into the cooling-room and kept there an hour or more, the temperature being lowered gradually till it is the same as in the body of the stable When this point is reached the horse is ready to be taken back to his stall, and is, to all intents, a new animal. If the first bath does not remove his complaint, he is given another and another on successive days till he is cured. The stablemen say that the horses have learned the pleasures of the Turkish bath, and evidently like the process. One horse, a very nice-looking gray, with a sore hind-leg, has taken four baths, and is said to walk up to the door of the bath-house whenever he is at liberty and wait to be let in. For strains, sprains, and various other ailments, electric baths are given. The horse to be treated with electricity is put in the warm room, and is well rubbed down with sponges attached to electric wires. The animals do not quite know what to make of the electric shock, but they are said to take kindly to it after they have had a little experience. In the Russian baths, where the steam is let out of the pipes into the room, till the atmosphere is wet enough to bottle, the horses sometimes rebel, but never make any very vigorous resistance. Several have been treated that were too sick to stand up in the bath. For the accommodation of such unfortunate invalids there is a stout blanket, fastened to ropes running through pulleys in the ceiling. The blanket is put under the horse's body, and, when the ropes are tightened, the horse enjoys the luxury of the bath swinging in a hammock. In every material point the baths are precisely similar to those provided for men and women. The horses are heated to a high point, deluged with cold water, thoroughly kneaded, slowly cooled off, and well rubbed down. The electric bath is a very powerful one, the batteries being capable of giving a shock that would make any human bather squirm.

Dr. Hough is enthusiastic in describing the results of his system of horse-baths. He tells of one of the best mares in the stables that two weeks ago had a very severe case of pneumonia. Her pulse was extremely high, and she had a dangerous rattling in the bronchial tubes. He put her in the new Russian bath and kept her in nearly all the afternoon, reducing the temperature gradually. She perspired very freely, and, when she was taken out of the bath, the rattling was entirely gone. The next day the mare was much better, and the bath was repeated. On the third day she was given another bath, and when she came out her pulse, respiration, and temperature were normal. At the end of a week she was entirely well and was put to work. For pink-eye, the baths are very efficacious. A horse was put in the hot-air bath with his eyes entirely closed and a heavy discharge of white mucus from eyes and nostrils. On the day following the first bath the swelling was greatly reduced and the discharge had disappeared. Two horses have been treated for founder, both successfully.

Cruelty to a Horse Punished.—Two dissected fetlock joints of a horse's feet were produced and offered in evidence in Special Sessions yesterday, before Justices Gardner, Morgan, and Smith, who were called upon to hear an extreme case of cruelty to animals. Officer Smart and Superintendent Hartfield, of the Society for the Prevention of Cruelty to Animals, arraigned at the bar John D. S. O'Brien and Henry Reising, both of Jamaica. Officer Smart swore that he arrested O'Brien on Sunday, December 18th, at Third Avenue and Twenty-second Street, while he was driving a team attached to a loaded hay-truck. One of the horses was suffering from shocking sores on both his fore feet, and walked on the back edges of his hoofs only. Both feet were swollen and bleeding, and it was evident that the animal could not have proceeded on its way but for the fact that the other horse partly dragged it along. The driver, Reising, who is also a stable-boy, told the officer that the horse had been harnessed that day at Rockaway Beach, and had been driven thence to this city. He was instructed to drive it by the prisoner, O'Brien, who owned it. The horse was subsequently subjected to examination by four veterinary surgeons, who found its feet in the last stages of suppuration and disease. The poor brute was then killed, and a post-mortem examination of the feet made for the purpose of securing proper evidence of the extreme and inhuman brutality of O'Brien in causing the horse to be worked at all. O'Brien's counsel did not attempt to deny the cruel treatment, but strove to have the case dismissed, on the ground that, as the horse had been harnessed in Rockaway, the case belonged to the jurisdiction of Queens County. The motion was denied and O'Brien was fined $150, the stable-boy, Reising, being fined $10.

Duck-Rearing in China. — Some years ago, Mr. George Brooke, the well-known poultry salesman of Leadenhall Market, showed me a letter from a gentleman detailing a system of duck-rearing which he had seen practiced in China. The method adopted was as follows: The ducks are hatched out by hot sand and divided into lots of a hundred to a hundred and fifty. A wicker fence is made to inclose a stream and its banks, the area of land and water inclosed being about twenty feet square; in this the young ducklings are placed. Mat sheds are made on the banks to shelter the ducklings from the heat of the sun or under which they may rest at night. They go to and from the stream or banks at their own will. A small boy takes charge of about five of these lots. When the ground gets foul, in the course of four or five days, the fence is moved much in the same way as hurdles are moved for sheep on turnips, three of the sides being shifted to inclose a fresh area adjoining. Large streams are made to branch off into several small ones, for the purpose of getting clean water to as many of the inclosures as possible. When very young, food is given them frequently during the day. It consists of boiled rice mixed with sweet potatoes, etc., and given them in the form of "dry dough." To prevent waste and to keep the soil clean, the food is strewn on mats. After the meal is over, the mats are washed and hung up to dry, which of course they do very quickly in a hot climate. There is very little food wasted, for the attendant knows almost exactly how much will suffice each lot for one meal. As the ducks grow, two lots of a hundred and fifty are put together, and a larger inclosure made; as they still further increase in size, two lots of three hundred are put together, and so on. Each time the inclosure is enlarged, the labor of one boy is dispensed with. The large lots require no more attention than the smaller ones, as the time of feeding is less frequent as the ducklings grow older. "When grown sufficiently strong, they are herded in flocks of some thousands by a man carrying a long bamboo rod, and he moves them from rice-field to rice-field, where they guddle amongst the mud, and are fed for almost nothing." They are brought home at night to sheds, which are floored with dry earth frequently changed and used as manure. The ducks when killed are, in most cases, "salted, smoked, and hawked about the streets of the large towns."

Toothsome Python Eggs.—When a German professor is engaged in what he believes to be scientific investigation his boldness knows no limits. Dr. Hermes, the learned Director of the Berlin Aquarium, did a deed in the interests of science only a few days ago that entitles him to rank among the most intrepid heroes of modern times. A "happy event" had recently come off in the reptile department of the Aquarium. No fewer than fifty-six eggs had been laid by one of the female pythons herein abiding. Dr. Hermes, on learning the joyful news, at once resolved to test the availability of serpentine ova for human consumption as an article of food. To this end he invited a select party of local gourmands to lunch, and commenced his experiments by boiling a python's egg for several minutes and offering it—opened in the usual manner—to his guests, who, however, respectfully declined to taste it, upon the ground that cooking had produced no visible alteration in the consistence of its external and internal substances—respectively a greenish leathery skin and an opaque, yolkless, gray liquid. Not particularly fancying the look of this egg himself, Dr. Hermes proceeded to open another, and emptied its contents into a frying-pan. Adding butter and salt in due proportions, he soon succeeded, with the aid of a gas stove, in producing a python omelet, which is reported to have "smelled uncommonly appetizing." Finally, none of his friends showing any ambition to vie with him for the honor of the first taste, he valiantly swallowed a mouthful, and pronounced it "supremely toothsome." Fired by his example all present then partook of the savory mess with apparent relish. Every one must admire Dr. Hermes's courage; but few probably will be disposed to emulate it. Pythons' eggs may be paramount delicacies. For the present, however, most people will persist in deriving the staple of their omelets from the farm-yard, in preference to the reptile house of the Zoo.

The British National Veterinary Congress.—Part I. of the "Proceedings of the British National Veterinary Congress," held in the rooms of the Society of Arts, in July last, has recently been published; and Part II, which is to contain the reports of committees and of colonial sections, will shortly follow in the form of an appendix. The project of assembling the members ot the veterinary profession for the discussion of urgent and important professional topics originated with a few veteriuarians attending the sittings of the British Medical Association held at Cambridge during August, 1880. The course of the discussion at the Public Health Section showed that much information in the possession of the veterinary profession required to be made generally public; and encouragement was derived from the fact that on the Continent, in various countries, gatherings of veterinarians had been recently tried on a large scale, and with marked success. Again, the profession in the United Kingdom had lately become consolidated by the admission of the Highland and Agricultural Society's graduates to the membership of the Royal College of Veterinary Surgeons; and, lastly, the proceedings at several meetings of veterinary societies had rendered it evident that some opportunity should be given of ventilating subjects of more than local professional importance, and on which the votes of a large number of members of the profession from different parts of the kingdom should be taken. The sittings of the Congress lasted two days, and the proceedings appear to have been satisfactory to the large number of member who attended it.

Influence of Race upon the Action of Poison.—The researches of M. Chauveau (*Jour. Therapeut.*) upon the relative immunity of Algerian sheep from malignant pustule, give support to this question. M. Bordier finds that the edible frog (Rana Esculenta) and the ordinary frog (Rana Temporaria) act very differently under the same quantity of *caffein;* whilst the tree frog (Rana Viridis) is less sensitive to the action of *veratrin* than the two preceding forms. In Tarentin, according to Darwin, the inhabitants only breed black sheep because the hypericum crispum, which abounds there, kills off all the white sheep within fifteen days. In Virginia the *lachnanthes tinctoria* destroys the white fowls, whilst black ones eat it with impunity. Cl. Bernard showed that different races of dogs and horses possessed distinctive physiological characteristics which were proportional to differences in the properties of certain histological elements, more especially in the nervous system. Negroes can take enormous doses of *tartar emetic*, and according to Dr. Thaly, they can ingest one gramme in the course of twenty-four hours with no more effect than would be produced by five cgr. in a white man. They also bear *mercury* well Broca also noticed that decomposition sets in more slowly in the bodies of persons belonging to this race. The negro can similarly carry a large quantity of *alcohol* without being overpowered by it; and in the black, white and yellow races an equal quantity of *alcohol* will not produce a similar state of intoxication. The yellow race can take large doses of drastic medicines.

The Salmon Disease.—Professor Huxley has a long paper in *Nature* describing the results of his recent researches into the disease which has during recent years played such havoc among the salmon in British rivers. He has made a thorough examination of the minute structure of both the healthy and diseased skin; and has tried some experiments on the transplantation of the *Saprolegnia* of the living salmon to dead animal bodies. In the latter case, the body of a recently killed common house-fly was gently rubbed two or three times over the surface of a patch of the diseased skin of a salmon, and was then placed in a vessel of water, on the surface of which it floated, in consequence of the large quantity of air which a fly's body contains. In the course of forty-eight hours, or thereabouts, innumerable white cottony filaments made their appearance, set close side by side, and radiated from the body of the fly in all directions. As these filaments had approximately the same length, the fly's body thus became inclosed in a thick white spheroidal shroud, having a diameter of as much as half an inch. As the filaments are specifically heavier than water, they gradually overcome the buoyancy of the air contained in the tracheæ of the fly, and the whole mass sinks to the bottom of the vessel. The following Professor Huxley gives as the chief results of this season's observations on the salmon disease. Incomplete as they are, they appear to justify the following conclusions: 1. That the *Saprolegnia* attacks the healthy living salmon exactly in the same way as it attacks the dead insect, and that it is the sole cause of the disease, whatever circumstances may, in a secondary manner, assist its operations. 2. That death may result without any other organ than the skin being attacked, and that, under these circumstances, it is the consequence partly of the exhaustion of nervous energy by the incessant irritation of the felted mycelium with its charge of fine sand, and partly of the drain of nutriment appropriated by the fungus. 3. That the penetration of the hyphæ of the *Saprolegnia* into the derma renders it at least possible that the disease may break out in a fresh-run salmon without reinfection. 4. That the cause of the disease, the *Saprolegnia*, may flourish in any fresh water, in the absence of salmon, as a saprophyte upon dead insects and other animals. 5. That the chances of infection for a healthy fish entering a river are prodigiously increased by the existence of diseased fish in that river, inasmuch as the bulk of *Saprolegnia* on a few diseased fish vastly exceeds that which would exist without them. 6. That, as in the case of the potato disease, the careful extirpation of every diseased individual is the treatment theoretically indicated; though in practice it may not be worth while to adopt the treatment.

A Hermaphrodite Hog.—The Nashville *Journal of Medieine and Surgery* reports a case of, as it claims, genuine hermaphroditism in a hog. The specimen was brought to the notice of the Secretary of the Savannah State Board of Health, and was carefully examined by Dr. J. E. Dobson. It consisted of a uterus having attached at one end an ovary, and at the other a well-developed testicle. Or rather the uterus, which in a hog is bifid, had only one end, while the other was an undeveloped scrotum. The testicle was perfect in all its appointments, having a spermatic cord and the usual folds attached. The outward appearance of the hog was that of a female, though the clitoris was evidently an imperfect penis, and the doctor had frequently noticed emissions of semen. Unfortunately for the cause of science the doctor failed to preserve all the attachments. It would have been peculiarly interesting to have noted the attachments of the spermatic cord, and it would have been well to have preserved all the genital organs. This is much to be regretted, as the clear case of a hermaphrodite is almost unknown. This is the best authenticated case we know of. It is evidently a commingling of the sexes in one being, as the uterus is only half developed, and there was but one ovary and one testicle. The hog had the habits of a male more than that of a female, though it was both one and the other. The specimen, in alcohol, was presented by Dr. Clark to the museum of the University of Nashville and Vanderbilt University.

Live Stock in the United States.—The census bulletin just issued giving the statistics of animals on farms in the United States, June 1st, 1880, shows that the increase of the stock since 1870 has been much greater than that of population, but a good deal less than the advance in the yield of corn. While the growth of population was 30 per cent. and of corn production 131, the rate of increase of horses was 45 per cent., milch cows 39, cattle 66, sheep 24, and swine 90. The increase of sheep is apparently less than that of population, but in reality it is believed to be greater, owing to the fact that the census returns of 1870 include a large number of spring lambs which are wholly excluded from the statistics of 1880. The leading wool-growing States are California with four and Ohio with five million sheep. But it is noteworthy that while the number has increased fifty per cent. in the former State it has decreased one per cent. in the latter. In Texas, Colorado, Nevada, and several of the Territories, there has been a most encouraging development of this industry. Texas is still the great cattle State, but in the rate of increase it has been surpassed by most of the other States. Iowa leads in swines, being credited with one-eighth of all in the United States, and showing the enormous increase of nearly 350 per cent. The magnitude of the dairy industry in New York is shown by the return of a million and a half cows, which is nearly double the number returned in any other State.

| | *Working Oxen.* | *Milch Cows.* | *Other Cattle.* |
|---|---|---|---|
| Total . . . . | 1,010,402 | 12,580,907 | 22,501,545 |
| Grand Total . . . . . . . . . | | | 36,093,854 |

| | |
|---|---|
| Sheep on farms in the United States . . . . | 35,191,656 |
| Ranch sheep . . . . . . . . . . . | 7,189,733 |
| Total . . . . . . . . . . . . | 42,381,389 |

—*Drovers' Journal.*

Dr. H. J. Detmers, sent to Texas by the Agricultural Department at Washington, is at present making his headquarters at San Antonio. His visit is an important one to the sheep and cattle interests of that State, as his mission is to investigate the diseases, their causes and remedy that prevails there. The lombriz in sheep is one of the most important points on which he wishes to report, and he asks as a special favor that any one that knows of the existence of lombriz, that he may be informed of it, and he will immediately proceed to the locality and endeavor to discover cause and cure for this scourge of the sheep men of South Texas. He also would be thankful for notice of any disease among imported sheep, as this is of great importance to the wool-growing industry. Spanish fever among cattle will claim a large share of his attention. With the assistance of the practical sheepmen of Texas, the doctor's visit can be made of great benefit to the industry, and we hope that they will lend every assistance possible. His address will be San Antonio, until further notice. Telegrams giving information as to the existences of urgent cases of disease can be sent at his expense.

PET ANIMALS AS CARRIERS OF CONTAGION.—The *Sanitary News*, calling attention to this danger, says: The contagious feature of some diseases has been disputed by the incredulous, because no channel of communication could be discovered between the first and subsequent cases. Dr. William Bunce, of Oberlin, O., reports a case in which five children of a family of six were taken with diphtheritic sore throat, no source of contagion being discoverable about the premises. A cat in the barn was found to have the characteristic lesions of diphtheria, and inquiry elicited the fact that the cat had been fondled by the children during its illness. A few months later, this same observer lost a patient from diphtheria, who had a few days before charitably removed some obstruction from the throat of a pet cat, which, upon examination, was found to be diphtheritic false membrane. In these instances the cats were made the carriers of the contagion by contracting the disease, either from some preëxisting case among animals of their own kind, or among people who petted them, or it may be from some unsanitary surroundings.

THE EARLY DEVELOPMENT OF FAST TROTTERS is an interesting fact in the physiology of growth. It is, no doubt, a fact that "baby trotters" do not, as a rule, last long. But there are some remarkable exceptions to this fact. The records now show that a yearling has trotted a mile in 2:42½, a 2-year old in 2:21, a 3-year old in 2:21, and a 4-year old in 2:19¾.

We give below a table showing the names, etc., of these trotters:

| *Name.* | *Desc.* | *Sire.* | *Rec'd.* |
|---|---|---|---|
| TWO-YEAR OLDS. | | | |
| Wildflower . . . | b. f. | Electioneer . . . | 2:21 |
| Fred Crocker . . | ch. c. | Electioneer . . . | 2:25½ |
| THREE-YEAR OLDS. | | | |
| Phil Thompson . . | g. g. | Red Wilkes . . | 2:21 |
| Sweetheart . . . | b. f. | Sultan . . . . | 2:22 |
| FOUR-YEAR OLDS. | | | |
| Trinket . . . . | b. m. | Princeps . . . . | 2:19¾ |
| Von Arnim . . . | b. s. | Sentinel . . . . | 2:22 |
| Romero . . . . | g. s. | A. W. Richmond . | 2.22½ |

STAMPING OUT DISEASE.—In Ottawa, Canada, it is announced that in consequence of the cattle disease, which has for some time existed in Pictou, N. S., the government has given the Minister of Agriculture power to send a veterinary surgeon to visit the places where the disease is said to exist, and power to declare any place where the disease is found an infected district. He will have power to order any cattle he thinks proper slaughtered, and no person but he, or some other person ordered by the department, will be permitted to remove any cattle from an infected district.—*National Live Stock Journal.*

A MONSTROSITY.—*The National Live Stock Journal* mentions a colt having been dropped at High Hill, Missouri, being well-formed except the head, which bore a remarkable rememblance to that of a human being. The upper part or top of the head was round, with prominent forehead; the opening for the eyes were placed as in a man, there were no eye-balls; the nose was a lump of loose skin, without opening; the upper jaw was short; the under jaw was shaped as in a man, but was longer, and had the under lip of the horse; it had no teeth; its ears were like those of a horse, and placed at the juncture of the head and neck. It lived about five hours, breathing through its mouth.

A FREAK OF NATURE is reported in the *Live Stock Journal* (Eng.) "of a calf with a perfect head of an elephant, including a trunk six inches in length. It is explained that the heifer that gave birth to the creature was very much alarmed at the sight of an elephant belonging to a traveling circus." A subscriber, not entirely convinced of the truth of the report, calls for further particulars, with name and address of owner, and adds, "What is *explained?* I suppose hundreds of pregnant heifers and cows have been frightened by locomotives, but I defy any of your readers, or or any one else, to give one well-authenticated case of a calf being born with a funnel and wheels in consequence of such fright."

